# Ring Finger Protein 11 Inhibits Melanocortin 3 and 4 Receptor Signaling

**DOI:** 10.3389/fendo.2016.00109

**Published:** 2016-08-08

**Authors:** Anne Müller, Lars Niederstadt, Wenke Jonas, Chun-Xia Yi, Franziska Meyer, Petra Wiedmer, Jana Fischer, Carsten Grötzinger, Annette Schürmann, Matthias Tschöp, Gunnar Kleinau, Annette Grüters, Heiko Krude, Heike Biebermann

**Affiliations:** ^1^Institut für Experimentelle Pädiatrische Endokrinologie, Charité-Universitätsmedizin Berlin, Berlin, Germany; ^2^Tumor Targeting Laboratory, Department of Hepatology and Gastroenterology, Molecular Cancer Research Center (MKFZ), Charité-Universitätsmedizin Berlin, Berlin, Germany; ^3^Department of Experimental Diabetology, German Institute of Human Nutrition Potsdam-Rehbruecke (DIfE), Nuthetal, Germany; ^4^German Center of Diabetes Research, Neuherberg, Germany; ^5^Department of Endocrinology and Metabolism, Academic Medical Center (AMC), University of Amsterdam, Amsterdam, Netherlands; ^6^Institut für Experimentelle Endokrinologie, Charité-Universitätsmedizin Berlin, Berlin, Germany; ^7^Institute for Diabetes and Obesity, Helmholtz Zentrum München, Germany, Deutsches Forschungszentrum für Gesundheit und Umwelt (GmbH), Neuherberg, Germany; ^8^Technische Universität München, München, Germany

**Keywords:** G protein coupled receptor, protein complementation assay, protein network, weight regulation, inflammation

## Abstract

Intact melanocortin signaling *via* the G protein-coupled receptors (GPCRs), melanocortin receptor 4 (MC4R), and melanocortin receptor 3 (MC3R) is crucial for body weight maintenance. So far, no connection between melanocortin signaling and hypothalamic inflammation has been reported. Using a bimolecular fluorescence complementation library screen, we identified a new interaction partner for these receptors, ring finger protein 11 (RNF11). RNF11 participates in the constitution of the A20 complex that is involved in reduction of tumor necrosis factor α (TNFα)-induced NFκB signaling, an important pathway in hypothalamic inflammation. Mice treated with high-fat diet (HFD) for 3 days demonstrated a trend toward an increase in hypothalamic *Rnf11* expression, as shown for other inflammatory markers under HFD. Furthermore, Gs-mediated signaling of MC3/4R was demonstrated to be strongly reduced to 20–40% by co-expression of RNF11 despite unchanged total receptor expression. Cell surface expression was not affected for MC3R but resulted in a significant reduction of MC4R to 61% by co-expression with RNF11. Mechanisms linking HFD, inflammation, and metabolism remain partially understood. In this study, a new axis between signaling of specific body weight regulating GPCRs and factors involved in hypothalamic inflammation is suggested.

## Introduction

Obesity and its associated comorbidities, such as type 2 diabetes or cardiovascular diseases, represent critical global health issues. Increasing caloric intake and/or reduced energy expenditure leads to excessive energy storage resulting in adipose accumulation, coupled to chronic low-grade inflammation (1–3) and weight gain. Numerous high-fat diet (HFD) analyses have revealed that inflammation not only occurs in peripheral tissue but also results in a non-classical form of inflammation in the hypothalamus, particularly in the arcuate (ARC) and paraventricular nucleus (PVN) (1, 3). Hypothalamic inflammatory changes have been detected within a short period of 3 days HFD (1, 4). In addition to microglia invasion, gene expression analyses revealed increased hypothalamic levels of inflammatory markers such as *interleukin 6* (*Il6*), *tumor necrosis factor α* (*Tnfα*), *suppressor of cytokine signaling 3* (*Socs3*), and *inhibitor of nuclear factor kappa-B kinase subunit beta* or *epsilon* (*Ikbkb/Ikbke*) (1, 4). The known functions of leptin and insulin receptors have been reported to be affected by HFD-induced hypothalamic inflammation. Hypothalamic leptin resistance has already been shown to develop very early in the first 1–3 days of HFD (5). Upregulation of *Socs3* and various inflammatory pathways are presumed to be contributors to leptin insensitivity (5, 6). Continuous HFD, inflammation, and manifesting leptin resistance lead to peripheral and hypothalamic insulin insensitivity (7).

Aside from leptin and insulin receptors, the G protein-coupled receptors (GPCR) and melanocortin 3 and 4 receptor (MC3/4R) are important regulators of body weight and energy consumption, as part of the hypothalamic leptin–melanocortin pathway (8).

Regarding the leptin–melanocortin axis, PVN expressed MC4R is the most prominent MCR in weight regulation (9, 10). MC4R knockout or loss-of-function variants in humans result in hyperphagia and early onset obesity (11–13). Furthermore, MC3R is expressed in the ARC on POMC neurons and agouti-related peptide (AgRP) neurons. MC3R knockout mice develop increased fat mass, hyperleptinemia, or hyperinsulinemia (14, 15). MC3/4R activity is primarily controlled by MSH, processed from POMC in anorexigenic POMC neurons and by AgRP, which is expressed in orexigenic AgRP neurons (10, 16, 17).

To further investigate the role of MC3R and MC4R, we applied a screening system that identified ring finger protein 11 (RNF11) as a potential hypothalamic melanocortin receptor interaction partner. RNF11 is a highly conserved E3 ligase, with different physiological functions (18) and operates as a compound of the A20 complex. The A20 complex is an ubiquitin-editing enzyme that plays a critical role in the termination of TNFα-induced canonical NFκB signaling pathway (19, 20).

In the present study, we tried to confirm the MCR/RNF11 interaction and analyzed the impact of RNF11 on MCR function in particular with regard to the early developed hypothalamic inflammatory changes during HFD.

## Materials and Methods

### Vector Construction

*Mc3r, Mc4r, Ghsr, Gpr83*, and *Rnf11* were amplified from murine hypothalamic cDNA. *MC3R* was amplified from human genomic DNA and *GHSR* from human cDNA (UMR cDNA Resource Center, Rolla, MO, USA). The pcDps [*NHA-Chrm3*], origin of all rCHRM3 (rat cholinergic receptor, muscarinic 3) constructs, was kindly provided by Torsten Schöneberg (University of Leipzig, Germany). The origin of YFP-fragment constructs was pcDNA3 (*MCFD2-YFP1/YFP2*) plasmids, which were kindly provided by Hans-Peter Hauri (University of Basel, Switzerland) (21). For direct interaction analysis of two known proteins in YFP-BiFc, *MCFD2* was replaced by the here relevant full-length genes (without stop codons). The pECFP-N1 (*Mc3r-YFP1*) (kanamycin resistance), required for the YFP-BiFc screening, was created by replacement of *ECFP* with *Mc3r-YFP1*. pECFP-N1 was purchased from Invitrogen (Darmstadt, Germany). The normalized murine hypothalamic cDNA library for screening was produced from wild-type hypothalami and inserted into pcDNA3-YFP2 (ampicillin resistance) by Eurofins MWG Operon (Ebersberg, Germany). The hemagglutinin tag was inserted at the amino terminus (NHA) and the FLAG tag at the carboxyl terminus. Reporter constructs pGL4.30 (*luc2P*/NFAT-RE/Hygro) and pGL4.33 (*luc2P*/SRE/Hygro) were purchased from Promega (Mannheim, Germany). The pGL4.34 (*luc2P*/NFκB-RE/Hygro) for detection of NFκB-activity was kindly provided by Vera Knaeuper (Cardiff University, Cardiff, United Kingdom).

### Cell Culture and Transfection

HEK293 and COS-7 cells were cultured as described elsewhere (22). For bimolecular fluorescence complementation (BiFc) approaches, HEK293 cells were seeded in 6-cm dishes (8.5 × 10^5^ cells/dish) and transfected with 1.9 μg DNA: 5.9 μl Metafectene^™^/dish (Biontex, Martinsried, Germany). For cAMP accumulation and cell viability tests, COS-7 cells were seeded into 96-well plates (0.9 × 10^4^ cells/well). For measurement of NFAT, SRE and NFκB activity *via* reporter gene assays, HEK293 cells were seeded in 96-well plates (1.5 × 10^4^ cells/well) coated with poly-l-lysine (Biochrom, Berlin, Germany). Cell viability of HEK293 cells was also examined in 96-well plates. Transfection in 96-well plates was performed with 41.7 ng plasmid DNA/well and 0.5 μl Metafectene^™^/well. For reporter gene assays, equal amounts of the appropriate reporter construct containing the firefly luciferase gene was co-transfected. For cell surface expression studies, COS-7 cells were seeded into 48-well plates (3.8 × 10^5^ cells/well) and transfected with 167 ng DNA and 1 μl Metafectene^™^/well (22). Total expression studies *via* sandwich ELISA were performed with COS-7 cells seeded in 6-cm dishes (6.5 × 10^5^ cells/dish) and transfected with 3 μg DNA and 8 μl Metafectene^™^/dish. All transfections were performed 1 day after seeding.

Cell lines that were chosen were well established in respective assays. YFP-BiFc analysis was performed in HEK293 cells due to optimal transfection results in combination with well detectable fluorescence signals. In addition to HEK293 cells, more robust COS-7 cells were used for wash intensive ELISA systems in expression studies. In regards to both cell lines, functional receptor studies were performed in HEK293 and COS-7 cells.

### Interaction Partner Screening and Association Studies *via* YFP-Based Bimolecular Fluorescence Complementation Assay

For screening, pECFP-N1 (*Mc3r-YFP1*) and pcDNA3 (*hypothalamic cDNA library-YFP2*) were co-transfected. Two days after transfection, cells were harvested and analyzed for fluorescence using AriaII SORP and the FACSDiva v6 program (Becton Dickinson, Heidelberg, Germany) at the Berlin-Brandenburg Center for Regenerative Therapies (BCRT, Charité Berlin). The cell sorter was adjusted with an interaction positive [MC3R-YFP1 + GHSR-YFP2 (23)] and negative control [rCHRM3-YFP1 + GHSR-YFP2 (24)]. Plasmid DNA from YFP-positive cells was purified using QIAmp DNA Blood Mini Kit (Qiagen, Hilden, Germany) and introduced into *E. coli* cells. Clones carrying library constructs were selected *via* antibiotic resistance, analyzed by sequencing, and evaluated *via* NCBI-BLAST.

Direct interaction or association between two defined proteins was analyzed in YFP-BiFc experiments using the full length genes as previously described (24). YFP-fluorescent cells were measured using the FACS Canto II at the BCRT and were analyzed with FlowJo 8.8.6 (Tree Star Inc., Ashland, OR, USA).

### Measurement of Signaling Pathways

Signaling pathways were analyzed 48 h after transfection. Intracellular cAMP accumulation for the determination of Gs activation was analyzed using the AlphaLISA technology (PerkinElmer, Rodgau, Germany). Human α-MSH was purchased from Sigma-Aldrich (Taufkirchen, Germany). Stimulation was performed for 45 min. Cell lysis (50 μl/well lysis buffer) and cAMP measurement were conducted as described elsewhere (22) according to the manufacturer’s protocol (PerkinElmer).

NFAT, SRE, and NFκB activity were determined in luciferase reporter gene assays. Stimulations with α-MSH, TNFα, and Ghrelin (Sigma-Aldrich) were performed for 6 h to allow appropriate reporter expression and according to the manufacturer’s instructions (Promega). Subsequently, cell lysis was performed with 50 μl/well of 1× Passive Lysis Buffer (Promega). Pathway activities were determined by luciferase activity according to the manufacturer’s protocol (Promega).

### Cell Surface and Total Expression Studies

Cell surface and total expression studies were performed in COS-7 cells 48 h after transfection using ELISA systems as previously described (22, 24). To investigate cell surface expression, HA-tagged receptors were required. For detection of the HA-tag, cells were washed, fixed with paraformaldehyde, and probed with a biotin-labeled anti-HA antibody (Roche Applied Science, Mannheim, Germany). Bound biotin anti-HA antibody was detected by peroxidase-labeled streptavidin (BioLegend, London, UK) in a substrate/chromogen reaction as previously described (25).

Total expression was measured using HA and FLAG double-tagged receptors. Cells were solubilized at 4°C [10 mM Tris/HCl, pH 7.4, 150 mM NaCl, 1 mM EDTA, 1 mM DTT, 1% sodium deoxycholat, 1% NP-40 and 0.2 mM PMSF (22)]. Lysates were incubated in anti-FLAG antibody (Sigma-Aldrich)-coated 96-well plates for 2 h. The HA epitope was detected as specified above. Total protein concentration of lysates was measured using a bicinchoninic acid (BCA)-based protein assay (Thermo Scientific, Bonn, Germany).

### Cell Viability Tests

Cell viability was measured using the CellTiter 96^®^ Aqueous One Solution Cell Proliferation Assay (Promega) according to the manufacturer’s instructions (10 μl 96^®^ solution/50 μl medium). Viability tests were performed 48 h after transfection.

### Animals and cDNA Synthesis

All procedures were approved by the Animal Care and Use Committee at the State Ministry of Rural Development, Environment and Consumer Protection of Brandenburg, Germany, in accordance with the German Animal Welfare Act (V3-2347-04-2013).

Hypothalami from *ad libitum* standard chow (S, 3.3 kcal% fat; Ssniff GmbH, Soest, Germany) and high-fat (HF, 60 kcal% fat; Research Diets Inc., New Brunswick, NJ, USA) diet-fed male mice (C57BL/6J) were sampled at an age of 6–6.5 weeks. HFD was administered for 3 days before salvation. RNA extractions were performed using 1 ml TRIzol^®^ reagent (Sigma-Aldrich) per hypothalamus according to the manufacturer’s instructions. Following DNaseI (New England Biolabs, Frankfurt am Main, Germany) digestion and before reverse transcription, the secondary structure of RNA was minimized by incubation at 60°C for 10 min. Synthesis of cDNA was performed using 400 ng RNA with 0.5 mM dNTP-Mix (Qiagen), 25 ng/μl Oligo(dT)15-Primer, and M-MLV Reverse Transcriptase under use of RNasin^®^ Plus RNase Inhibitor (Promega).

### Quantitative Real-time PCR

Quantitative real-time PCR was performed using the SsoFast^™^ EvaGreen^®^ Supermix (Bio-Rad, Hercules, CA, USA). Primers specific for six reference genes were analyzed for the compared murine samples: *ribosomal protein, large, P0 (Rplp0), 18S ribosomal RNA (Rn18S), beta-actin (Actb), polymerase (RNA) II (DNA directed) polypeptide A* (*Polr2a*), *TATA box binding protein* (*Tbp*), and *hypoxanthine guanine phosphoribosyl transferase* (*Hprt*). The reference gene *Actb* was chosen in this experiment due to its minimal variation across experimental groups (analyzed by geNorm of the qBase software, Zulte, Belgium). Quantitative PCR data were analyzed using the comparative Ct method. Ct is the quantification cycle number at which the light outcome of qPCR is first recorded above the background signals. The difference of the average Ct value for the gene of interest and the average Ct value for the reference gene represents ΔCt. To receive the relative quantitation of gene expression, the arithmetic formula 2^−ΔCt^ was used (Applied Biosystems’ User Bulletin 2, Foster City, CA, USA).

### Statistical Analyses

All data were checked for normal distribution and expressed as mean ± or + SEM. Statistical analyses were performed using the statistical tools implemented in Graph Pad Prism, version 6 (GraphPad Software, San Diego, CA, USA).

## Results

### RNF11 as Screening Hit for a Potential MC3R Interaction or Association

In the initial screening *via* YFP-based bimolecular fluorescence complementation (YFP-BiFc), we investigated potential new murine hypothalamic MC3R interaction partners. The MC3R was chosen due to its known and particularly well-characterized capability to interact in a functional relevant manner with several other proteins such as receptors and accessory proteins (23, 26, 27). The initial sorting process was stopped after 30 min. During this time, 3,900 YFP fluorescent cells were collected. Subsequent selection for library constructs resulted in 239 bacterial clones. Sequence analysis, in which false positive hits were sorted out (e.g., cDNA fragment not in frame with YFP fragment, cDNA fragment consisting of non-coding DNA region), revealed 16 potential hypothalamic interaction partners for the murine MC3R. The detected potential interaction partners were proteins that are typically found in multiple protein complexes or involved in trafficking processes, cell metabolism, protein degradation, cell–cell communication, and cellular nutrient sensing (Table S1 in Supplementary Material). In the present study, we focused on the interplay with RNF11. RNF11 plays a crucial role in reducing NFκB signaling and, therefore, in the counterregulation of inflammation. RNF11 represents an important component of the NFκB-inhibiting A20 complex (19, 20). NFκB, which is activated by TNFα, has been shown to result in an upregulation of A20 expression and in a higher interactivity of RNF11, assembling the A20 complex and protecting cells from constant NFκB signaling (20). Known hypothalamic expression, present structural protein-binding motifs (28, 29), and previously published interaction findings (19, 20) give rise to the possibility that RNF11 represents a potential and important interaction partner for neuronal GPCRs of weight regulation.

In addition to MC3R, we also concentrated on the most important GPCR in hypothalamic weight regulation, the MC4R. MC3R and MC4R share a high sequence similarity. Therefore, further analyses focused on both melanocortin receptors.

### RNF11 Interacts with MC3R and MC4R

**Figure 1:**
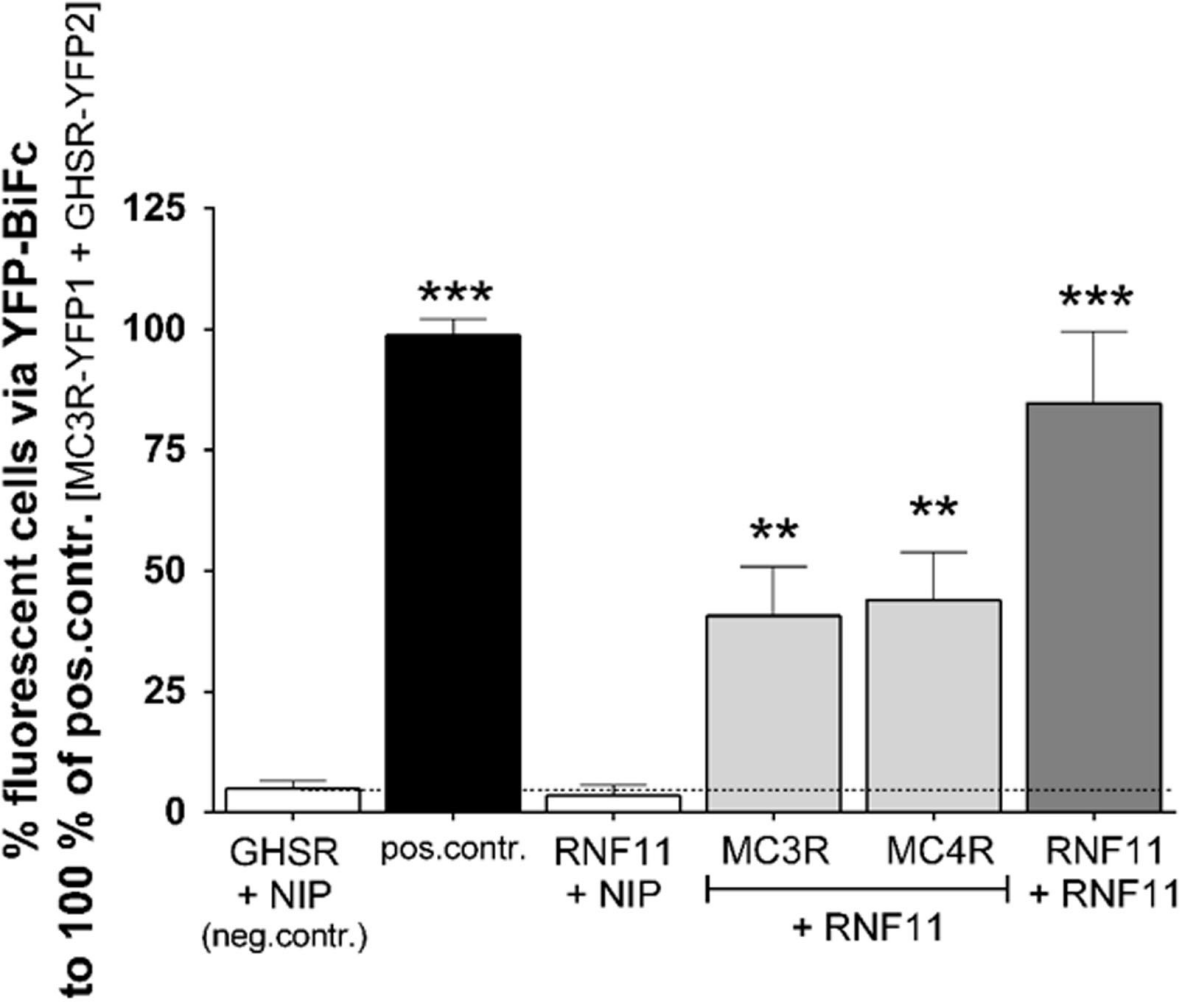
**RNF11 interacts with MC3/4R**. Interaction studies *via* YFP-based protein complementation assay (YFP-BiFc). Positive control, human MC3R/GHSR heterodimer (23); negative control, GHSR/non-interacting protein (NIP, rCHRM3) co-expression (24). No YFP1/YFP2 tags are displayed due to the combination of values from *vice versa* assays (e.g., first gray column: values from RNF11-YFP1 + MC3R-YFP2 and values from RNF11-YFP2 + MC3R-YFP1 experiments). Fifty thousand cells were analyzed on fluorescence per sample and experiment. Data were assessed from a minimum of three independent experiments, each performed in triplicates, and calculated to the control. Values represent the mean + SEM. Significance to negative control ***p ≤ 0.001 (one-way ANOVA, Dunnett’s test).

To confirm MC3R/RNF11 interaction and further examine RNF11/MC4R interplay, YFP-BiFc studies with full-length genes were performed (Figure 1). The co-expression of GHSR and a non-interactive protein (NIP) represented by the rat cholinergic receptor, muscarinic 3 [rCHRM3 (24), Figure 1, first white column] served as interaction negative and the MC3R/GHSR heterodimer as interaction positive controls for GPCRs [(23), Figure 1, black column]. Furthermore, previous studies of YFP-BiFc have shown that GPCRs do not randomly aggregate with any other co-expressed and overexpressed proteins in this assay system (24). Interaction negative controls for MC3/4R (co-expression with NIP, rCHRM3) have been previously shown (24, 30), and similar results were reported as the negative control in the present study. To establish RNF11 interaction controls, homodimerization of the soluble protein was demonstrated (Figure 1, dark gray column) in addition to non-interaction with rCHRM3 (Figure 1, third column). Interaction of RNF11 with MC3/4R was confirmed *via* full-length YFP-BiFc (Figure 1, light gray columns).

In BiFc studies, fluorescence analysis does not provide quantitative information of intracellular interaction as it is usually irreversible. Equal expression of transfected YFP1 and YFP2 tagged proteins was assumed but not verified. Furthermore, due to the detection of interactions within a distance of several nanometers by BiFc, interaction partners that act imbedded in complexes and direct protein–protein interactions can be identified using BiFc. In a sandwich ELISA system including destructive cell lysis and several washing steps, RNF11 interactions were not detected. Therefore, we cannot exclude that further cellular proteins are involved in, or even mediate, the MC3R and MC4R interaction with RNF11. For simplification purposes, the MCR/RNF11 interplay will be termed here as interaction.

### MC3/4R Signaling Capacity Is Inhibited by RNF11

**Figure 2:**
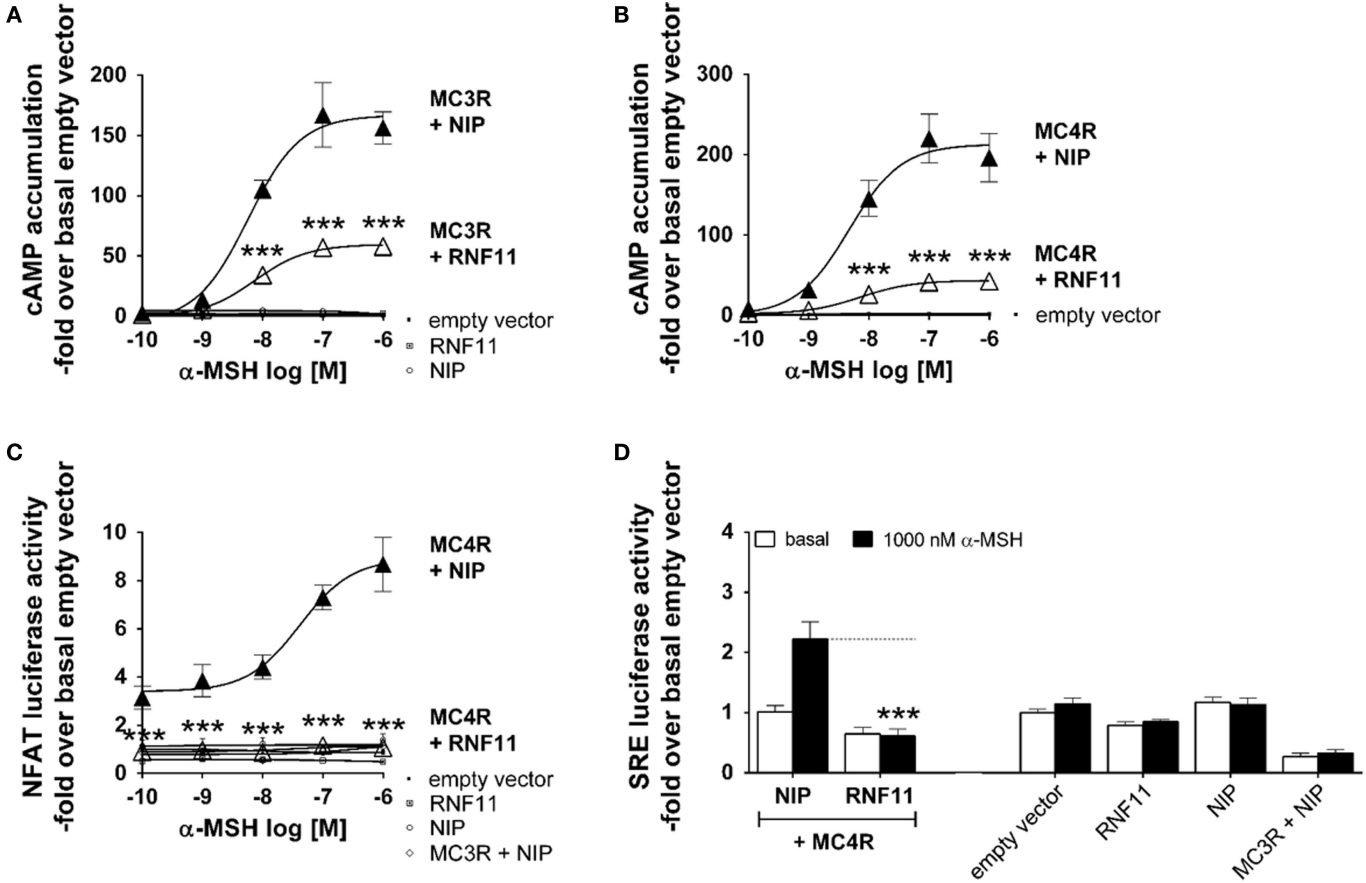
**RNF11 inhibits MC3/4R signaling**. Functional studies of MC3R and MC4R in cAMP accumulation **(A,B)**, NFAT **(C)**, and SRE-controlled luciferase activity **(D)**. Concentration response curves to α-MSH **(A–C)**. MC3/4R co-expressions with a non-interactive protein [NIP, rCHRM3 (30); black filled triangles] are opposed to the GPCR/RNF11 interplay (unfilled triangles). In **(D)** MC4R, co-expression with NIP (first column pair) is opposed to the MC4R/RNF11 interaction (second column pair) in the absence (basal) or presence of α-MSH. For assay control, single gene or plasmid transfections of the empty vector, RNF11, and NIP were performed. To ensure equal amounts of plasmid DNA in each transfection, single gene approaches were filled with empty vector. Data were assessed from a minimum of three independent experiments, each performed in triplicates. Values represent mean ± SEM. Significance was determined as stimulated MCRs + NIP to stimulated MCRs + RNF11. ***p ≤ 0.001 (two-way ANOVA for factor RNF11 and α-MSH, Sidak test due to extreme low SDs in co-expressions with RNF11 compared with MCRs/NIP).

Alpha-MSH-induced cAMP accumulation (Gs signaling) through MC3R (Figure 2A) and MC4R activation (Figure 2B) in co-expression with the NIP [rCHRM3 (24, 30)] resulted in typical concentration response curves with a stimulation maxima of 160 to 200-fold cAMP accumulation compared with the basal empty vector. EC50 values of MCR/NIP co-expressions were measured at 5–8 nM α-MSH (Figures 2A,B). Co-expression with RNF11 led to a reduction to 44% of MC3R signaling and 19% of MC4R signaling in Gs in response to 100 nM α-MSH without an effect on the EC50 value (Figures 2A,B). In addition to the main Gs pathway, ligand-induced activation of MC4R has been reported to modify the activity of extracellular regulated kinases [(31, 32) ERK 1/2 signaling], AMP-activated kinases (33), c-jun kinases (34), protein kinases C (35), and phosphatidylinositol-3-kinases [(31, 36) IP3 formation]. To investigate whether RNF11 specifically inhibits Gs signaling of MCRs, two additional pathways were analyzed (Figures 2C,D). The activity of the transcription factor NFAT and therefore NFAT-driven luciferase activity (Figure 2C) occurs downstream of IP3 formation. SRE-driven luciferase activity (Figure 2D) suggests an upstream MAPK/ERK 1/2 activity. The murine MC4R in co-expression with NIP showed a 3.1-fold basal NFAT luciferase activity compared with the empty vector control (Figure 2C). Furthermore, stimulation with α-MSH also resulted in a distinct concentration response curve with a maximum 8.6-fold of NFAT luciferase activity compared with the basal empty vector with an EC50 value of 70 nM α-MSH (Figure 2C). Co-expression of RNF11 resulted in a complete loss of MC4R-mediated NFAT-driven luciferase activity (Figure 2C). In the SRE pathway (Figure 2D), no basal activity of MC4R/NIP co-expression was detected. Stimulation with 1000 nM α-MSH led to a slight 2.2-fold increase in SRE luciferase activity compared with the basal empty vector, supporting previous data that ERK 1/2 mainly results from Gq/11 (IP3 formation) (31) (Figure 2D). Consistently, also for SRE signaling, RNF11 co-expression diminished MC4R-mediated signaling in total as reported for NFAT activity (Figures 2C,D). For the murine MC3R, neither basal nor α-MSH stimulated NFAT or SRE luciferase activity was detected (Figures 2C,D). The expression of the empty vector, RNF11 and NIP (rCHRM3) alone (served as controls), did not show basal activity or stimulation by α-MSH in the investigated signaling pathways (Figure 2).

### MC3/4R Expression Profiles Are Differently Modulated by RNF11

**Figure 3:**
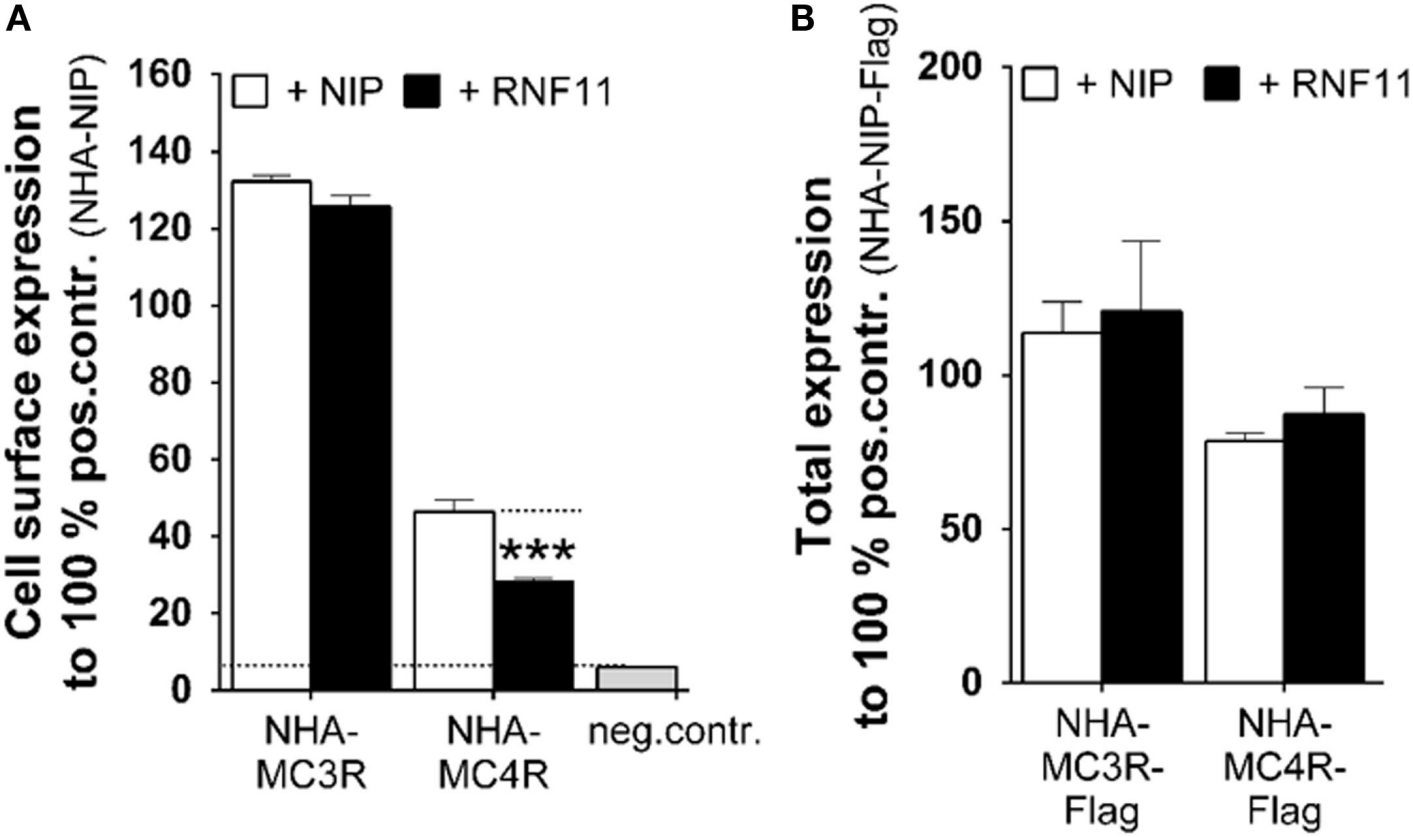
**Expression of MC3/4R in the presence of RNF11 is differentially influenced**. **(A)** Cell surface expression and **(B)** total expression of MC3/4R was analyzed in ELISA systems. The negative control in both expression studies was untagged rCHRM3 (NIP). Positive controls were NHA-rCHRM3 [NHA-NIP **(A)**] and NHA-rCHRM3-FLAG [NHA-NIP-Flag **(B)**]. MC3/4R co-expression with NIP (white columns) is opposed to the GPCR/RNF11 interplay (black columns). In cell surface expression studies, GPCRs were detected *via* the N-terminal HA. In total expression analysis, the HA- and Flag-double tagged receptor was used. Data were assessed from a minimum of three independent experiments, each performed in triplicates and calculated to 100% of the positive control. Values represent mean + SEM. Significance was determined as NHA-MC4R + NIP compared to NHA-MC4R + RNF11. ***p ≤ 0.001 (unpaired *t*-test, two tailed).

Cell surface and total expression studies were performed (Figures 3A,B). Using this approach, we aimed to investigate whether the observed functional influence on MC3/4R in the presence of RNF11 (Figure 2) was due to aberrant receptor expression. Co-transfection with NIP (Figures 3A,B, white columns) was compared with MC3R/RNF11 or MC4R/RNF11 co-transfections (Figures 3A,B, black columns). Cell surface expression of MC3R was not significantly reduced by RNF11 (Figure 3A). However, the cell surface presence of MC4R was significantly reduced to 61% by co-expression with RNF11 (Figure 3A). Total receptor expression of both analyzed MCRs was not modified by co-expression with RNF11 (Figure 3B).

**Figure 4:**
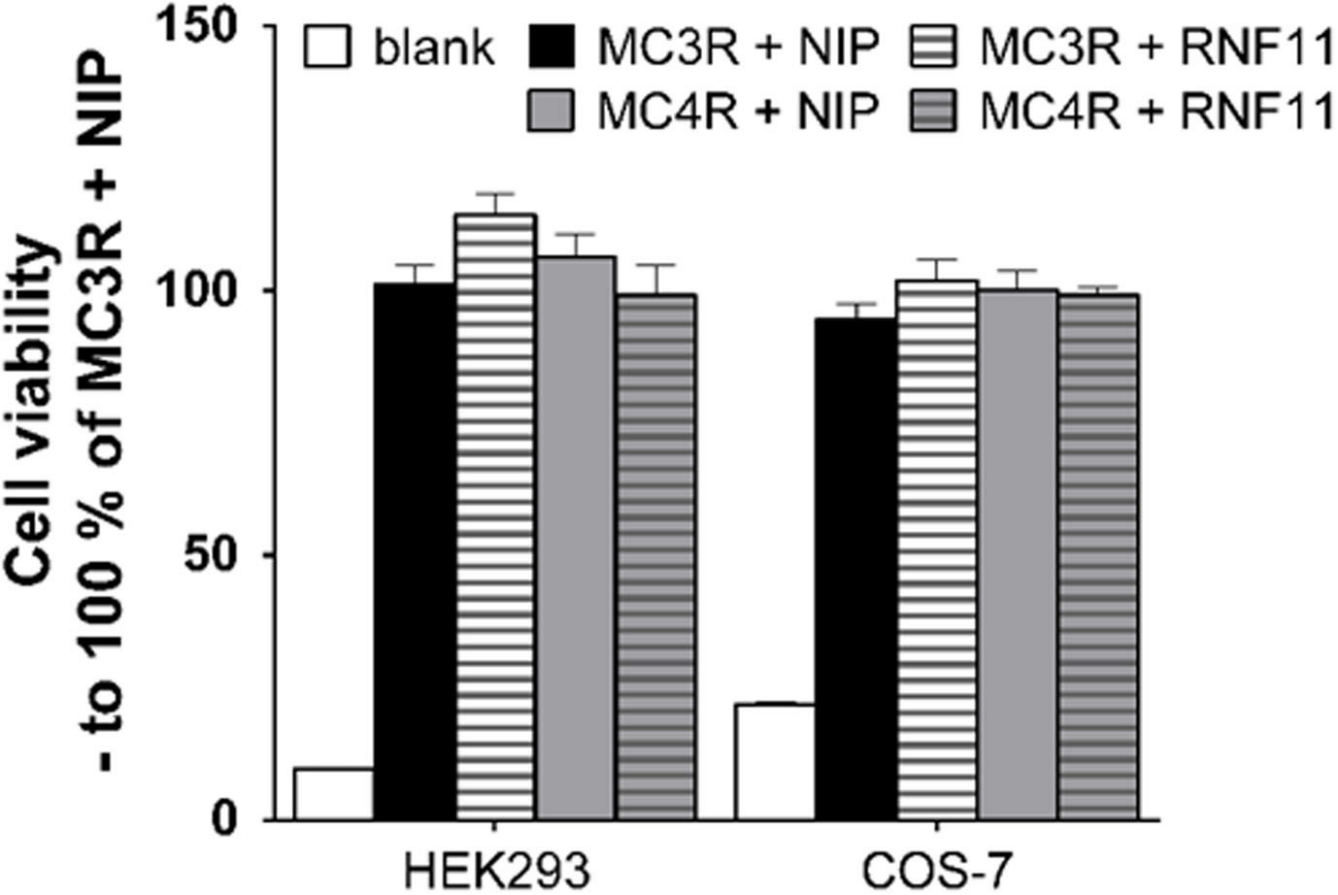
**Cell viability is not influenced by RNF11 expression**. Cell viability during MC3/4R and NIP expression (black and gray columns) was compared with MC3/4R and RNF11 expression (striped columns) in the used cell systems. Data were assessed from a minimum of three independent experiments, each performed in triplicates and calculated to 100% control. Values represent mean + SEM.

To analyze whether the decrease of GPCR expression and function in the presence of RNF11 is due to increased cell death, cell viability was investigated in HEK293 and COS-7 cells, corresponding to interaction, cAMP accumulation, reporter gene assays, or expression studies. As depicted in Figure 4, cell viability was not affected by RNF11 expression.

### RNF11 Has a Greater Impact on NFκB Signaling Reduction than α-MSH

**Figure 5:**
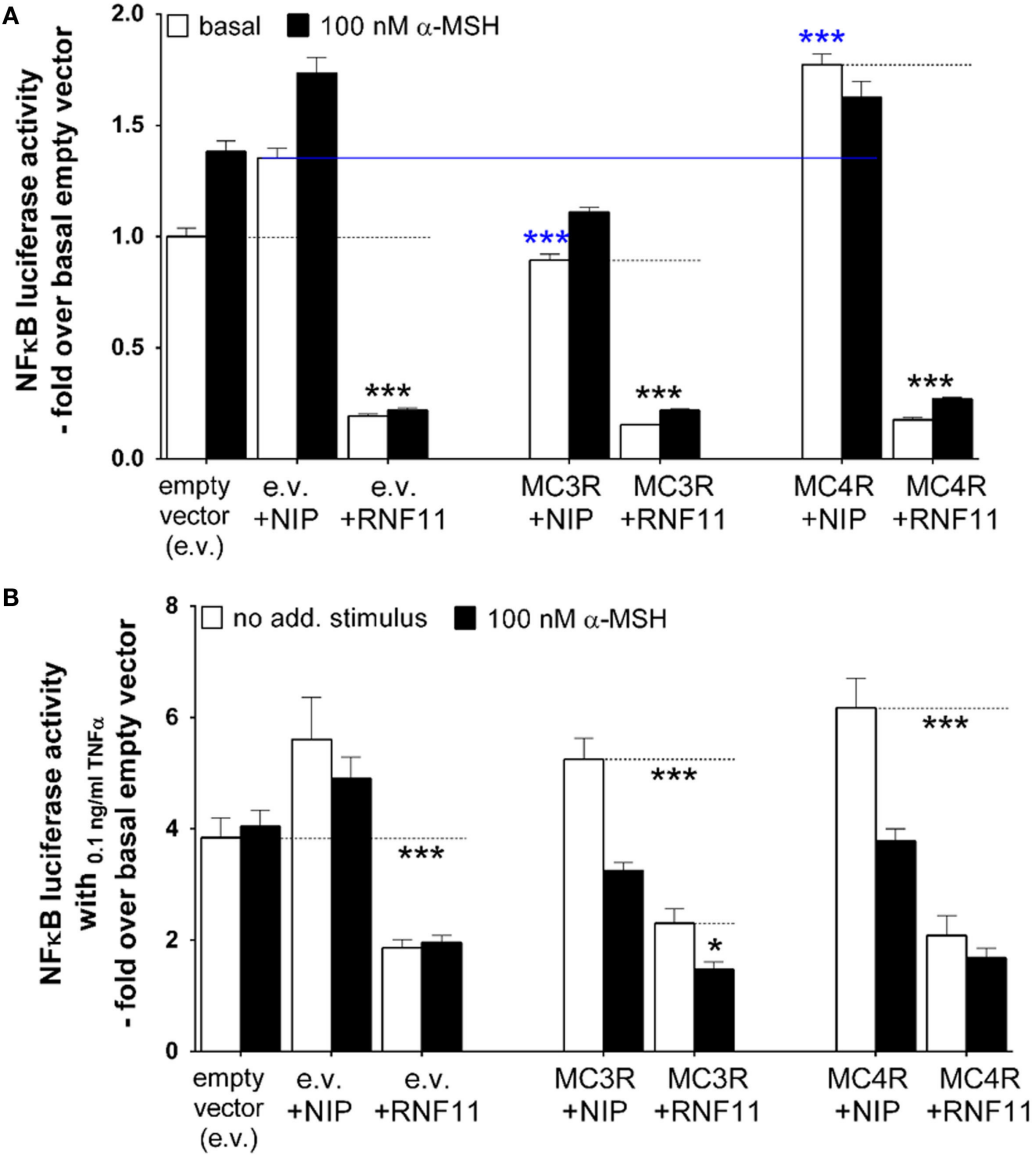
**RNF11 has stronger impact on decreasing NF**κ**B signaling than stimulated MC3/4R**. NFκB was measured using a luciferase reporter gene assay. In **(A)**, basal NFκB (white columns) is compared with NFκB activity during α-MSH stimulation (black columns). In **(B)**, cellular NFκB activity was stimulated with 0.1 ng/ml TNFα. In comparison, NFκB signaling in absence (white columns) and presence of α-MSH (black columns). MC3R or MC4R in co-expression with the non-interactive protein [NIP, rCHRM3 (30)] is opposed to MC3/4R interactions with RNF11. The first three column pairs [empty vector (e.v.), e.v. + NIP, and e.v. + RNF11] served as controls. Data were assessed from a minimum of three independent experiments, each performed in triplicates, and calculated fold over the basal empty vector. Values represent mean + SEM. Significance determined by one-way ANOVA and Dunnett’s test: basal e.v. compared with e.v. + RNF11 **(A,B)**; basal e.v. + NIP compared with basal MC3/4R + NIP [**(A)**, blue statistic]; basal MC3/4R + NIP to MC3/4R + RNF11 **(A,B)**; Significance determined by unpaired *t*-test, two-tailed: α-MSH effect on MC3/4R + NIP **(B)**; α-MSH effect on MC3R + RNF11 **(B)**. *p ≤ 0.05; ***p ≤ 0.001.

In addition to analysis of MC4R signaling and expression in interaction with RNF11, the influence of MC4R on RNF11 controlled action was also examined. Therefore, NFκB signaling was investigated by measuring NFκB-driven luciferase activity (Figure 5). NFκB luciferase activity was monitored in the absence (white columns) and presence (black columns) of 100 nM α-MSH (Figure 5) and without (Figure 5A) or with (Figure 5B) additional NFκB stimulation by TNFα. The first three column pairs of empty vector, NIP (rCHRM3), and RNF11 represent controls (Figure 5).

Data demonstrated clearly the reported NFκB inhibiting effect of RNF11 expressed alone (19, 20) compared with the empty vector alone, basal (Figure 5A), as well as under the 0.1 ng/ml TNFα-stimulated condition (Figure 5B). Furthermore, basal NFκB signaling (Figure 5A) was slightly yet significant reduced by MC3R and marginally enhanced by MC4R (MC3/4R in co-expression with NIP compared with NIP alone). Although alpha-MSH had no additional decreasing effect on the basal NFκB state, RNF11 also inhibited NFκB signaling in co-expression of MC3/4R almost completely (Figure 5A). Upon TNFα stimulation (Figure 5B), the reported NFκB inhibiting effect of α-MSH on melanocortin receptors [(37) MC3/4R co-expressed with NIP] was observed. A NFκB reduction of approximately 39% was documented (Figure 5B). However, RNF11 expression alone and in combination with MC3/4R also resulted in the TNFα-stimulated background to a stronger NFκB abatement than MSH. In all cases, NFκB signaling was reduced to approximately twofold over the basal empty vector by RNF11 (Figure 5B, reduction of 50–70%). Interestingly, for MC3R/RNF11 co-expression, a slight synergistic effect of NFκB inhibition by RNF11 and α-MSH was observed, which indicates that basal NFκB signaling of MC3R + RNF11 is decreased compared with MC3R + NIP. A further decreasing effect in NFκB is obtained by α-MSH stimulation in MC3R + RNF11 co-expression (Figure 5B).

### RNF11 Expression Trends toward an Increase after 3 Days of HFD

**Figure 6:**
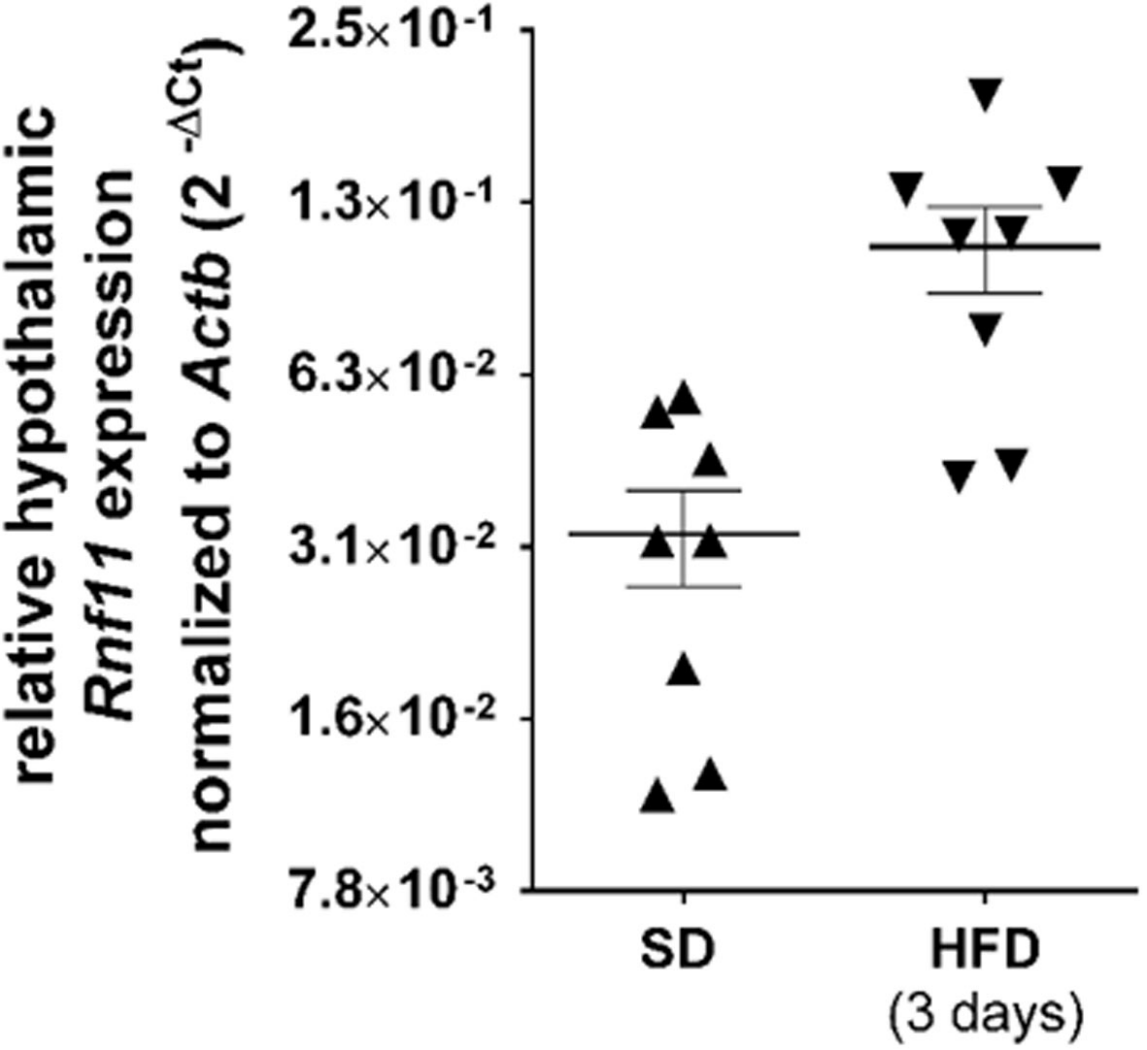
**Relative hypothalamic *Rnf11* expression trends toward an increase following 3 days of HFD**. Each group comprised of eight animals. qPCR of SD and HFD cDNA samples was performed in the same reaction plate. Gene expression of animals was analyzed in duplicates. All symbols display the mean of duplicates for one animal (detailed data can be found in Table S2 in Supplementary Material). For each diet group, the mean ± SEM between the animals is depicted. Scale of *Y*-axis represents Log2. Reference gene, *beta-actin* (*Actb*); SD, standard diet; HFD, high-fat diet.

The present data implies that RNF11 may play a role in the inhibition of MCR function during inflammation. Therefore, we analyzed whether hypothalamic NFκB inhibiting protein RNF11, as part of the A20 complex, is upregulated after 3 days of HFD, comparable to NFκB increasing inflammatory factors (1). As various genes are altered in expression after a short-term period of nutritional change (38), expression variation of six housekeeping genes were analyzed among standard diet and HFD-fed animals (see Materials and Methods). Only *beta-actin* was observed to be not affected by 3-day HFD and was used as reference gene in quantitative expression studies. Quantitative real-time PCR demonstrated *Rnf11* expression in HFD-treated animals to be threefold higher than in mice that were fed a standard diet, although this was not significant (Figure 6).

Future studies should focus on whether methods other than BiFc can be used to analyze RNF11/MCR interaction. As Sandwich ELISA approaches failed to detect this interaction in the present study, we speculate that other proteins mediate the functional relevant RNF11/MCR interaction. Therefore, mass spectrometry may be applied whereby whole protein complexes are extracted in an initial step. In an additional step, RNF11 and MC3R or MC4R may be detected by tag-specific antibodies. Furthermore, more physiological cell lines such as hypothalamic cells are preferable for *in vitro* studies. In the present study, the hypothalamic neuronal cell lines N41, N39, and GT1-7 were analyzed; however, we were unable to obtain consistent results due to poor transfection efficiency or expression level. Still, in the conducted *in vitro* studies, RNF11 and MC3/4R are comparably overexpressed. Further studies have to reveal the physiological *in vivo* relevance of the results.

## Discussion

### RNF11 Influences MC3/4R Activity *via* Different Mechanisms

Functional characterization of MC3/4R revealed reduced signaling of these GPCRs due to co-expression with RNF11 (Figure 2). Furthermore, signaling inhibition was not restricted to the main MCR cAMP accumulation pathway, but was also constrained to the other analyzed pathways. Therefore, RNF11 appears to be a universal signaling blocking factor on MCRs. Further expression studies demonstrated that the RNF11 effect on MC3/4R is most likely generated by two different mechanisms.

(I) RNF11 can inhibit cell surface expression of a certain GPCR, as demonstrated in this study for MC4R, contributing to limited signaling capacity. Binding partners of RNF11 that have been identified so far suggest that RNF11 is likely to be involved in numerous signaling and trafficking processes (39). For example, RNF11 directly interacts with proteins of the ubiquitination machinery (ITCH, NEDD4, and UBC13) and with proteins mediating transport processes [e.g., GGA family of clathrin adaptors (40)]. RNF11 controls ubiquitination and thereby the function of GGA proteins, which have been shown to be involved in GPCR cell surface transport through direct interaction (41). Consistently, we demonstrated the ability of RNF11 to reduce MC4R cell surface expression, without changing total expression levels, which may be due to internalization or retaining of receptors in the endoplasmic reticulum and Golgi (Figures 3A,B).
(II) Analyses of the MC3R, which reside at the cell surface after RNF11 co-expression (Figure 3A), lead to the hypothesis that the association of RNF11 or RNF11-related protein complexes may inhibit GPCR activity by interfering with G protein coupling or G protein subunit release during activation.

These facts indicate specific actions of RNF11 on different GPCRs, resulting in the reduction of signaling capability. Several recognition sites on GPCRs, presented in different stages of receptor maturation, may determine favored RNF11 actions.

In addition to the RNF11 effect on MC3/4R function observed here, we also tested the impact of RNF11 on other hypothalamic GPCRs (Figure S1 in Supplementary Material). An interaction of RNF11 with the growth hormone secretagogues receptor (GHSR) and the G protein-coupled receptor 83 (GPR83) could be shown in addition to a signaling inhibition for both GPCRs through RNF11 co-expression. These data indicate a very consistent general action of RNF11 on GPCR function.

### RNF11 Function on Hypothalamic Melanocortin Receptors and Its Potential Role in Inflammation

**Figure 7:**
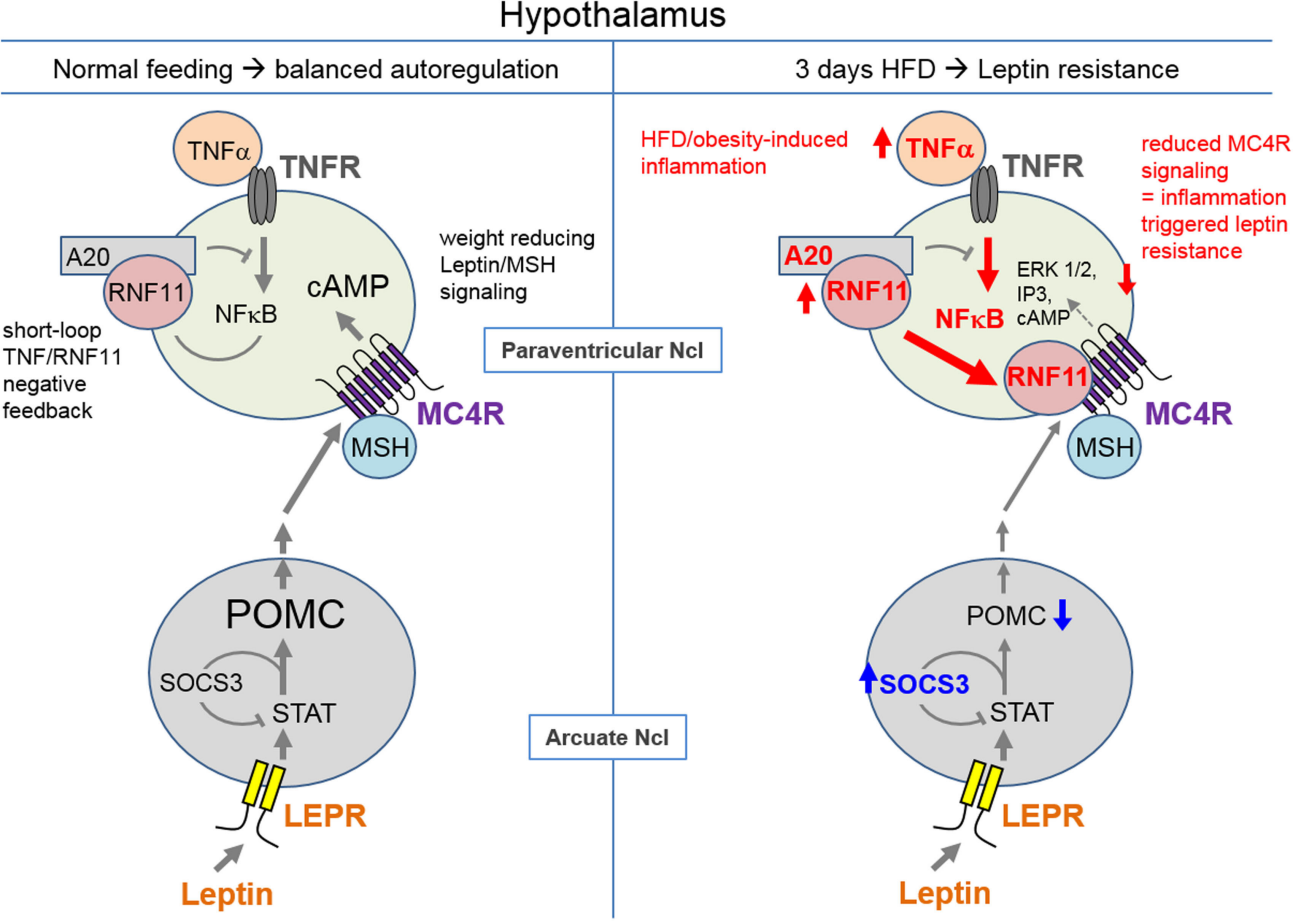
**Hypothesis of how MC4R/RNF11 interplay may result in early development of leptin resistance during high-fat diet**. Under healthy conditions (left side), leptin, which is produced in adipocytes, acts on its receptor, which is (LEPR) located on anorexigenic pro-opiomelanocortin (POMC) neurons. POMC is processed to melanocyte-stimulating hormones (MSH), which activate the weight controlling MC4R in its signaling pathways. NFκB signaling based on tumor necrosis factor α (TNFα) acting on TNFR (TNF receptors) is also found on neurons. For both, POMC and NFκB production negative feedback loops exist. STAT signaling of LEPR leads to SOCS3 expression and decreased LEPR signaling. TNFα/NFκB results in A20 complex formation and decreased NFκB signaling. RNF11 is part of the A20 complex. Hypothalamic inflammation and leptin resistance characterized by elevated TNFα (and subsequently A20) and SOCS3 have been reported (1, 4, 46) after 3 days of high-fat diet (HFD, right side). Furthermore, RNF11 is upregulated (Figure 6) contributing to leptin resistance and obesity by direct inhibition of MC4R signaling (Figure 2). Ncl, nucleus.

Chronic non-regulated inflammation is observed in the pathogenesis of obesity, diabetes, arteriosclerosis, Alzheimer’s disease, and gout. Previously, numerous different factors such as acetylcholine (42), adenosine (43), or peptides, such as melanocortins (44, 45), have been identified to play a role in inflammation regulation. The targets of these factors are primarily GPCRs. For the first time, the present study indicates that RNF11 (as part of the A20 complex) represents a further factor that interacts with GPCRs. We speculate this interaction may be relevant in hypothalamic inflammation. NFκB, activated by TNFα, has been shown to lead to upregulation of A20 expression and to a higher interactivity of RNF11 for the assembly of the A20 complex, protecting the cell from constant NFκB signaling (20) in an auto-regulatory manner (Figure 7). By identifying RNF11 as an interaction partner of MC3/4R, we hypothesize that RNF11 potentially links hypothalamic inflammation to MC3/4R signaling, a characteristic that has so far been attributed to leptin and insulin receptors. So far, *Rnf11* knockout animals are not existent, probably due to lethality by the loss of the multifunctional protein.

### Altered Hypothalamic RNF11 Expression during the First Day of HFD May Contribute to Leptin Resistance and Obesity Manifestation

Obesity and a HFD, even after 3 days (1), are associated with systemic low-grade inflammation and leptin resistance on the molecular signaling level. Circulating inflammatory cytokines such as TNFα, free fatty acids, and immune cells reach brain structures particularly at the hypothalamic level to provoke local inflammation [(47, 48), Figure 7]. Located at the third ventricle, and therefore exhibiting areas in which the blood–brain barrier is loosened, the hypothalamus, in particular the ARC, can be effectively reached by peripheral messengers. Peripheral inflammatory markers have been shown to activate microglia cells that produce local inflammatory cytokines such as TNFα, in turn promoting a secondary hypothalamic inflammation process (3, 49). Hypothalamic inflammation, characterized by elevated TNFα or microglia activity, has been documented after a short-term HFD (1).

One prominent inflammatory pathway activated by TNFα is NFκB. NFκB activity can be found on neurons and microglia and is clearly associated with obesity [(50, 51), Figure 7]. Activation of NFκB in microglia has been shown to lead to the degradation of, for example, dopaminergic neurons (52). Hypothalamic RNF11 appears to be upregulated in combination with A20 (Figure 7) to immediately counteract HFD-induced NFκB (Figure 6) and to provide protection particular to neurons. However, we hypothesize that higher RNF11 expression and activity increases the probability of MCR/RNF11 interaction, leading to desensitization of these GPCRs within 3 days of HFD (Figure 7). A decrease in the function of MC3R and particularly MC4R strongly contributes to the development of leptin resistance and obesity manifestation [(11, 13), Figure 7]. Moreover, the very early loss of MC4R function leads to increased food and caloric intake (11). Thus, RNF11 represent a further molecular detail how hypothalamic inflammation may trigger leptin resistance and obesity in the very early days of high caloric intake (Figure 7).

Our RNF11 expression studies were performed with complete hypothalami, including many different cell types (3). It would be of interest to observe whether significant RNF11 upregulation can be specifically found at the ARC and PVN neurons after 3 days of HFD.

Furthermore, hypothalamic expression and activity of RNF11 may potentially be regulated in a time-dependent manner. Constant RNF11 upregulation would block NFκB signaling and MC3/4R or receptor resensitization, which may be of pathophysiological relevance.

In summary, RNF11 was presented as a new molecular link of how HFD exposure may result in functional melanocortin dysfunction. RNF11, which was observed to be marginally upregulated within the first 3 days of HFD feeding, inhibited MC3/4R function in *in vitro* studies. This lead to the hypothesis; that reduction of hypothalamic MC4R signaling, shortly after initiation of a HFD, may explain the early onset of functional leptin resistance, which contributes, in part, to further manifestations of obesity (Figure 7).

## Author Contributions

AM performed most of the experiments, analyzed the data, and wrote the manuscript. LN conducted the real-time PCR analysis and discussed the data. WJ and PW realized the feeding experiments, removed the hypothalami from animals and revised the work critically. C-XY, FM, JF, and GK helped in different experiments, contributed to the progression of the work and discussed the data. CG, AS, MT, AG, HK, and HB designed and supervised the project, enabled the experiments, analyzed and interpreted the data and wrote the manuscript.

## Conflict of Interest Statement

The authors declare that the research was conducted in the absence of any commercial or financial relationships that could be construed as a potential conflict of interest.
