# Supplementary material for: Ring Finger Protein 11 Inhibits Melanocortin 3 and 4 Receptor Signaling
Source: Front Endocrinol (Lausanne). 2016 Aug 8;7:109. doi: 10.3389/fendo.2016.00109 (PMC4976663; doi:10.3389/fendo.2016.00109)
Supplement: Supplementary file 1 [file Table_1.DOCX]

Supplementary Material

**Ring finger protein 11 inhibits melanocortin 3 and 4 receptor signaling**

Anne Müller, Lars Niederstadt, Wenke Jonas, Chun-Xia Yi, Franziska Meyer, Petra Wiedmer, Jana Fischer, Carsten Grötzinger, Annette Schürmann, Matthias Tschöp, Gunnar Kleinau, Annette Grüters, Heiko Krude and Heike Biebermann*

*** Correspondence:** Heike Biebermann: heike.biebermann@charite.de

TABLE S1 *Potential MC3R interaction partners identified by bimolecular fluorescence complementation*

| **Function** | **Gene symbol** |
| --- | --- |
| complex associated | *Wdr82, Snrp* |
| trafficking processes | *Dnm3, Snap25, Tbc1d19* |
| cell metabolism | *Enpp5, Pkm2, Pfkm, Golm1* |
| protein degradation | *Rnf11, Ube2v1, Hecw1* |
| cell-cell communication | *Celsr3, Apmap* |
| cellular nutrition-sensing | *Glut1, Fnip* |

**
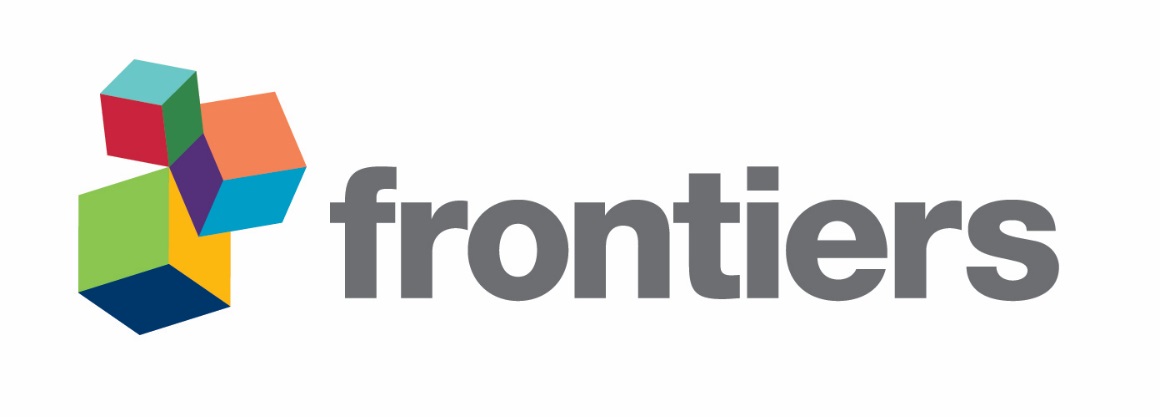
**
