# Supplementary material for: Ring Finger Protein 11 Inhibits Melanocortin 3 and 4 Receptor Signaling
Source: Front Endocrinol (Lausanne). 2016 Aug 8;7:109. doi: 10.3389/fendo.2016.00109 (PMC4976663; doi:10.3389/fendo.2016.00109)
Supplement: Supplementary file 2 [file Table_2.DOCX]

Supplementary Material

**Ring finger protein 11 inhibits melanocortin 3 and 4 receptor signaling**

Anne Müller, Lars Niederstadt, Wenke Jonas, Chun-Xia Yi, Franziska Meyer, Petra Wiedmer, Jana Fischer, Carsten Grötzinger, Annette Schürmann, Matthias Tschöp, Gunnar Kleinau, Annette Grüters, Heiko Krude and Heike Biebermann*

*** Correspondence:** Heike Biebermann: [heike.biebermann@charite.de](mailto:heike.biebermann@charite.de)

TABLE S2 *Duplicates of quantitative real-time PCR*

| **Animals, fed standard (SD) or high-fat diet (HD, for 3 days)** | **Hypothalamic *Rnf11* expression in duplicates, normalized to *beta-actin* (2^-ΔCt^)** | |
| --- | --- | --- |
| SD1 | 0,051890 | 0,062690 |
| SD2 | 0,019810 | 0,044370 |
| SD3 | 0,051870 | 0,037520 |
| SD4 | 0,018410 | 0,019960 |
| SD5 | 0,006980 | 0,016130 |
| SD6 | 0,013870 | 0,011250 |
| SD7 | 0,030390 | 0,033640 |
| SD8 | 0,046210 | 0,061410 |
| HFD1 | 0,079390 | 0,138680 |
| HFD2 | 0,133830 | 0,128790 |
| HFD3 | 0,079440 | 0,141610 |
| HFD4 | 0,072040 | 0,312800 |
| HFD5 | 0,025830 | 0,056830 |
| HFD6 | 0,125760 | 0,142640 |
| HFD7 | 0,039760 | 0,046950 |
| HFD8 | 0,078150 | 0,071190 |

**
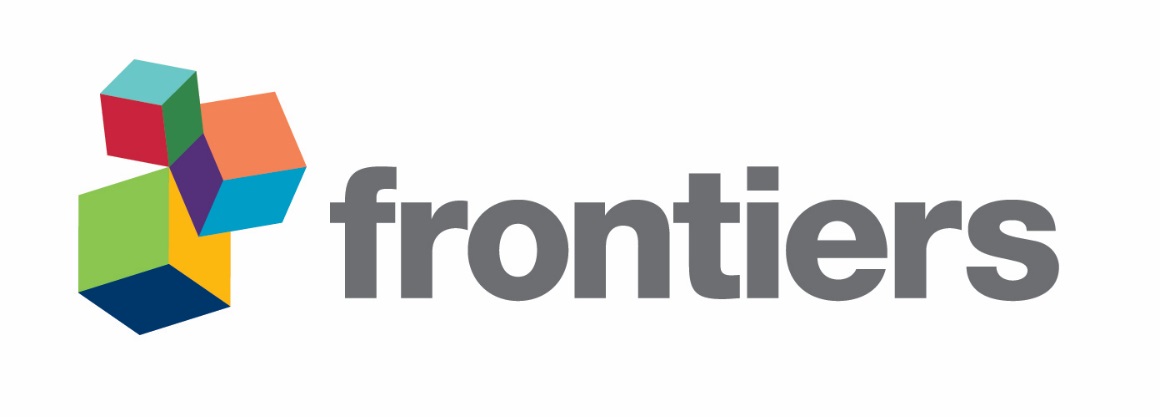
**
