# Supplementary material for: Ring Finger Protein 11 Inhibits Melanocortin 3 and 4 Receptor Signaling
Source: Front Endocrinol (Lausanne). 2016 Aug 8;7:109. doi: 10.3389/fendo.2016.00109 (PMC4976663; doi:10.3389/fendo.2016.00109)
Supplement: Supplementary file 3 [file Image_1.PDF]

Supplementary Material

Ring finger protein 11 inhibits melanocortin 3 and 4 receptor signaling

Anne Müller, Lars Niederstadt, Wenke Jonas, Chun-Xia Yi, Franziska Meyer, Petra Wiedmer, Jana Fischer, Carsten Grötzinger, Annette Schürmann, Matthias Tschöp, Gunnar Kleinau, Annette Grüters, Heiko Krude and Heike Biebermann*

*** Correspondence:** Heike Biebermann: heike.biebermann@charite.de

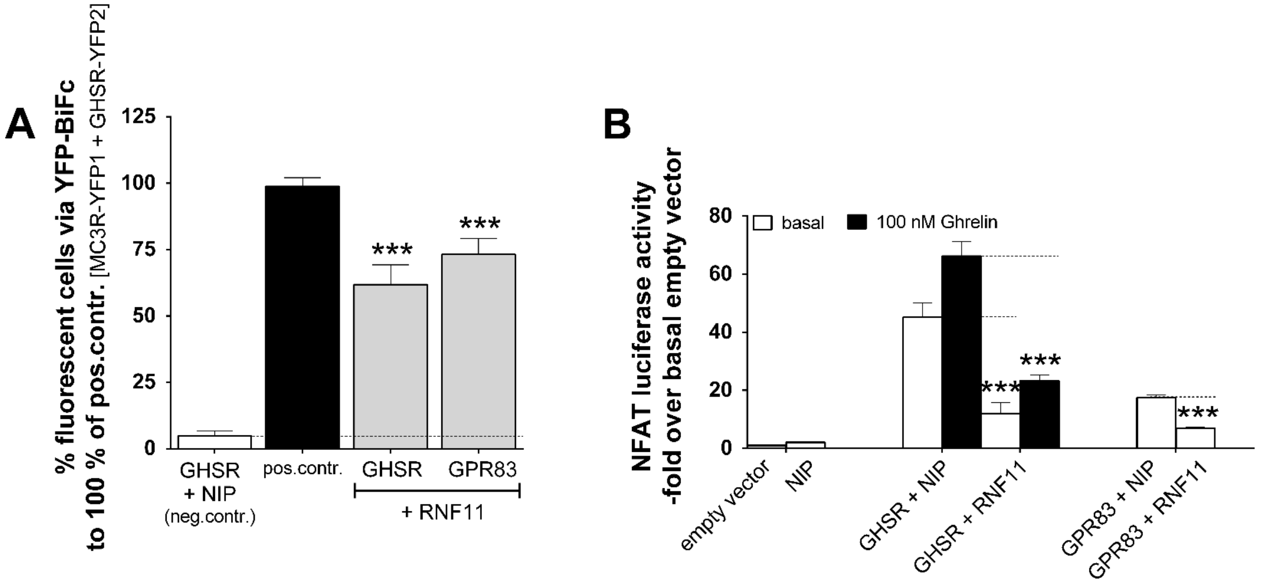

FIGURE S1 | RNF11 interacts with the murine GHSR and GPR83 and inhibits receptor signaling. (A) Interaction studies via YFP based protein complementation assay (YFP-BiFc). Positive control (pos.contr.): human MC3R/GHSR heterodimer, negative control (neg.contr.): GHSR/non-interacting protein (NIP, rCHRM3) co-expression. No YFP1/YFP2 tags are displayed due to the combination of values from *vice versa* assays (e.g. first grey column: values from RNF11-YFP1 + MC3R-YFP2 and values from RNF11-YFP2 + MC3R-YFP1 experiments). Per sample and experiment 50.000 cells were analyzed for fluorescence. **(B)** Functional analysis of NFAT controlled luciferase activity. GHSR and GPR83 co-expressions with a non-interactive protein [NIP, rCHRM3] are opposed to the GPCR/RNF11 co-expressions. GHSR was additionally stimulated with 100 nM ghrelin. Three independent experiments, performed in triplicates. Values represent mean + SEM. *** $p \leq 0.001$ (A, one-way ANOVA, Dunnett´s test; B, unpaired t-test, two-tailed).
